# Supplementary material for: The Construction and Exploration of a Comprehensive MicroRNA Centered Regulatory Network in Foxtail Millet (Setaria italica L.)
Source: Front Plant Sci. 2022 May 6;13:848474. doi: 10.3389/fpls.2022.848474 (PMC9121102; doi:10.3389/fpls.2022.848474)
Supplement: Supplementary file 4 [file Data_Sheet_4.zip › Sit-miR172a_Seita.2G091100.1_1500_TPlot.pdf]

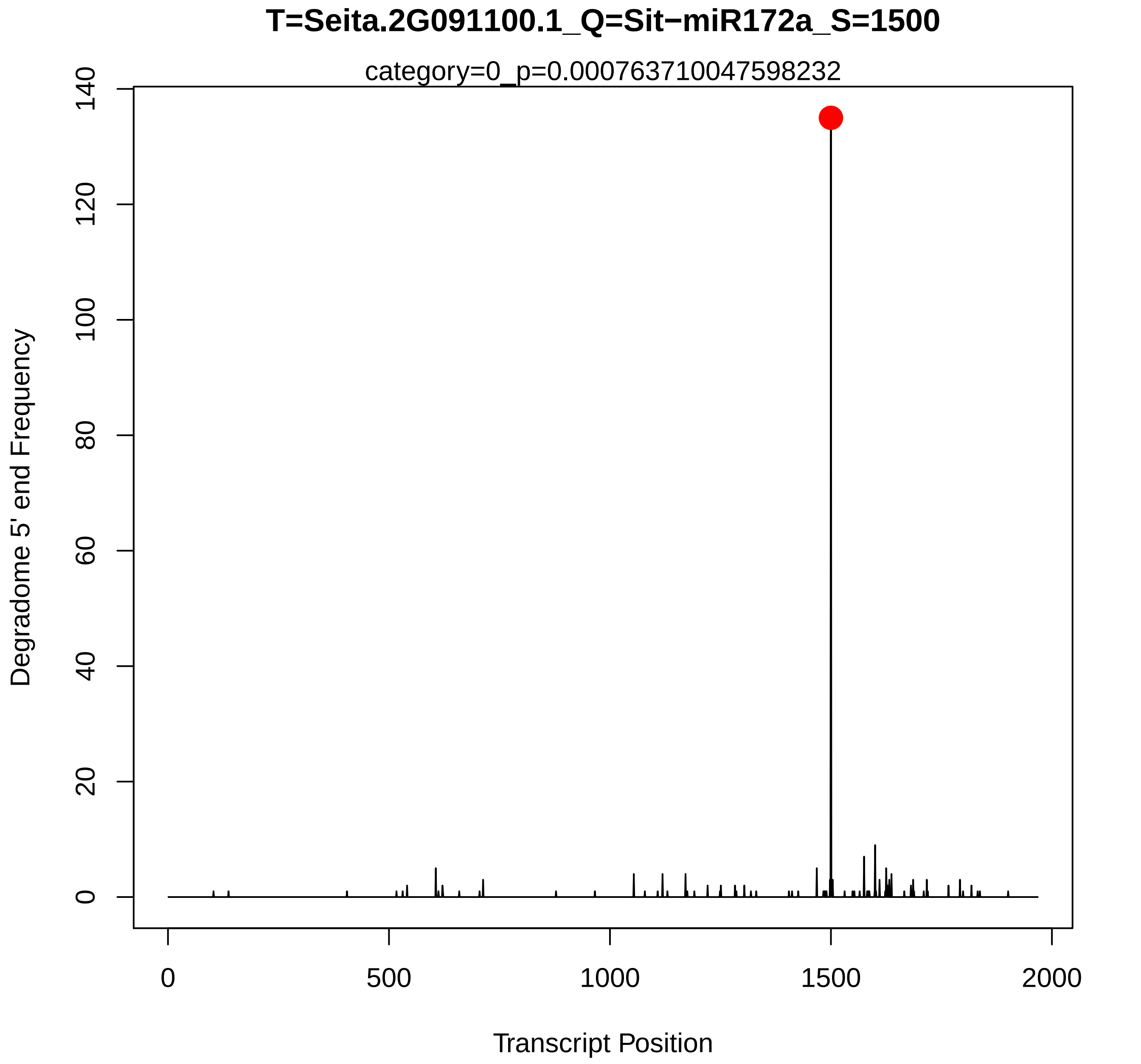

T=Seita.2G091100.1_Q=Sit−miR172a_S=1500
category=0_p=0.000763710047598232
Degradome 5' end Frequency
Transcript Position
0
20
40
60
80
100
120
140
0
500
1000
1500
2000
